# Association between daily life experience and psychological well-being in people living with nonpsychotic mental disorders: Protocol for a systematic review and meta-analysis

**DOI:** 10.1097/MD.0000000000009733

**Published:** 2018-01-26

**Authors:** Jeffery Ho, Shirley P.C. Ngai, William K.K. Wu, Wai Kai Hou

**Affiliations:** a Department of Anesthesia and Intensive Care; b Li Ka Shing Institute of Health Sciences, The Chinese University of Hong Kong, Shatin; c Department of Rehabilitation Sciences, The Hong Kong Polytechnic University, Hung Hom; d Laboratory of Psychology and Ecology of Stress (LoPES); e Department of Psychology; f Centre for Psychosocial Health, The Education University of Hong Kong, Tai Po, Hong Kong.

**Keywords:** anxiety, daily life experience, depression, life satisfaction, nonpsychotic, post-traumatic stress, psychological well-being

## Abstract

**Background:**

Evidence has shown that people living with nonpsychotic mental disorders experience difficulties in maintaining their daily living, consequently impacting on psychological well-being. However, the role of daily life experience remains unclear. This systematic review and meta-analysis aims to determine the association between daily life experience and psychological well-being in people living with nonpsychotic mental disorders, and evaluate daily life experience as a moderator of psychological well-being in this population.

**Methods:**

Literature search will be performed using a combination of title/abstract words and subject headings on 7 electronic databases according to predefined inclusion and exclusion criteria. Data will be extracted by 4 independent reviewers (JH, SPCN, WKKW, and WKH). Disagreement will be resolved by discussion with senior reviewers. Observational studies involving subjects with unipolar depression, bipolar disorder, anxiety disorder, acute stress disorder, as post-traumatic stress disorder as distinct groups with quantitative measurement of daily life experience and psychological well-being will be included.

**Results:**

Effect sizes will be pooled by random effects model. The quality of the studies will be assessed using Newcastle–Ottawa scale. Heterogeneity between studies will be quantified using *I*^2^ index. This review is registered in PROSPERO.

**Conclusions:**

While symptoms and existing treatments of nonpsychotic mental disorders could be long term and dependent upon medical regimens, sustaining daily life experience will be a potentially important and concrete pathway that empowers patients to recover from the disorders, maintain or enhance psychological well-being, and be reintegrated into society. Findings of this review will inform prospective interventional trials of enhancing daily life experience in prevention of recurrence and enhancing psychological well-being in people living with nonpsychotic mental disorders.

## 1 Introduction

Nonpsychotic mental disorders are associated with significant morbidity and burden of the society. These encompass unipolar depression, bipolar disorder, anxiety disorder, acute stress disorder, and post-traumatic stress disorder (PTSD).^[1]^ According to the World Health Organization, nonpsychotic mental disorders are responsible for one-third of years of life lost due to disability (YLD) worldwide. Of these, bipolar disorders and unipolar depressive disorders account for 295 and 1500 per 100,000 population worldwide, respectively. Notably, the burden of these affective disorders ranked first for bipolar (89 per 100,000) and second for unipolar depressive disorders (390 per 100,000) in Western Pacific Region. The burden of depressive disorders ranked top in Southeast Asia, with 410 per 100,000) are living with this disease.^[2]^ These collectively led to 24.3 millions of YLD (8.3% of total YLD) in male and 41.0 millions (13.4% of total YLD) in female globally, representing the top cause of YLD of all human diseases. Nevertheless, this analysis of YLD did not take into account of quality of life, and psychological well-being of individuals other than loss of health.^[2]^ In general, psychological well-being refers to a state of life satisfaction, life engagement, and positive self-recognition.^[3]^

Evidence has shown that people living with nonpsychotic mental disorders are prone to daily life stress, consequently impacting on psychological well-being.^[4–7]^ A community-based survey revealed that people living with nonpsychotic mental disorders were more likely to report stress (odds ratio = 1.8, 95% CI 1.1–2.8) and poorly perceived mental health status (OR = 25.6. 95% CI 14.7–45.0).^[8]^ More clarity is nonetheless needed in conceptualizing and assessing everyday life processes in affective disorders and PTSD. Daily stressors could be either caused or worsened by traumatic experiences or affective symptoms. In addition, previous studies assessed and aggregated a wide variety of daily stressors including activities or behaviors (e.g., home repairs, shopping), social partners (e.g., children, spouse), relationships (e.g., problem getting along with fellow workers), thoughts and feelings (e.g., having free time, being lonely), generic conditions (e.g., weather, physical environment), or even objects (e.g., television). Some of these “daily stressors” are indeed ordinary daily life experiences, stress-impacted daily life experiences, or obstacles to sustaining them. In-depth investigation on mental health could be limited without teasing apart variations in the impacts of different everyday life processes that contextualize adaptation.^[9,10]^

However, the role of daily life experience remains unclear. This systematic review and meta-analysis aims to determine the association between daily life experience and psychological well-being in people living with affective disorder, and evaluate daily life experience as a moderator of psychological well-being in people living with affective disorder.

## 2 Methods

### 2.1 Literature search and study selection

We will conduct literature search on electronic databases including PubMed, (MEDLINE), PsycINFO, Cumulative index to nursing and allied health literature (CINAHL), Scopus, Web of Science, and Excerpta medica DataBase (EMBASE). Relevant articles will be identified using Medical Subject Heading (MeSH) or title/abstract keywords from inception to August 2018 without language restriction. The search terms comprise keywords related to: (1) affective disorders, (2) acute stress and post-traumatic stress disorders, (3) anxiety disorders, (4) psychological well-being, and (5) daily life experience. Details of the search strategies have been summarized in Table 1. All citations will be imported into Endnote X8 software by which duplicated will be removed. Four researchers (JH, SPCN, WKKW, and WKH) will perform the article screening according to the inclusion and exclusion criteria. Studies preliminarily included in this stage will be cross-checked by other independent reviewers. Any disagreement in study selection will be resolved by discussion with senior authors. This study will be conducted according to Preferred Reporting Items for Systematic Reviews and Meta-Analyses guidelines published in 2009.^[11]^ This protocol is registered in PROSPERO, an international prospective register of systematic reviews at the National Institute for Health Research and the Center for Reviews and Dissemination (CRD) at the University of York. This review protocol is reported according to the Preferred Reporting Items for Systematic Review and Meta-analysis Protocols (PRISMA-P) 2015 checklist.^[12]^

**Table 1:**
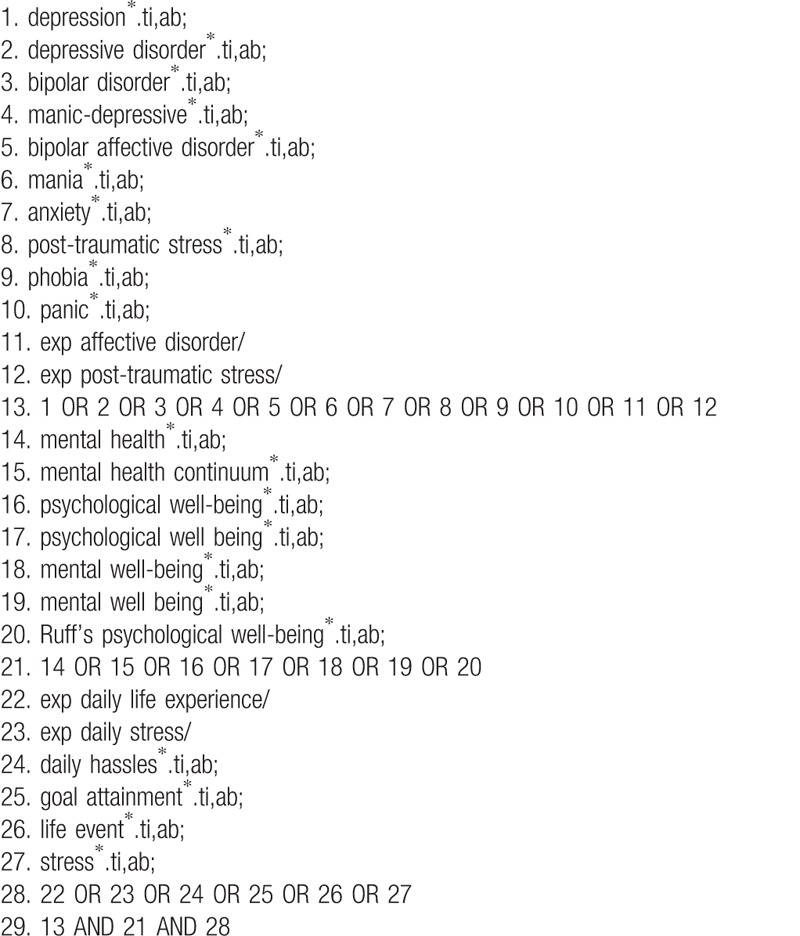
Search strategies.

### 2.2 Inclusion and exclusion criteria

Observational studies with quantitative measurement of daily life experience and psychological well-being in people living with nonpsychotic mental disorders will be included. Study participants with bipolar disorders, dysthymia, major depressive disorders, manic-depression, anxiety disorder, acute and post-traumatic stress disorders should be identified as distinct groups. The diagnosis of nonpsychotic mental disorders should have been confirmed by the following well-established diagnostic criteria: Diagnostic and statistical manual of mental disorders (DSM) 4th edition or International Statistical Classification of Diseases (ICD) and related health problems version 9 or 10. Studies on participants with self-reported mental disorders or without defined participants will be excluded. Qualitative inquiries, interventional studies, and nonoriginal articles are not eligible. The details of the inclusion and exclusion criteria for this systematic review are summarized in Table 2.

**Table 2:**
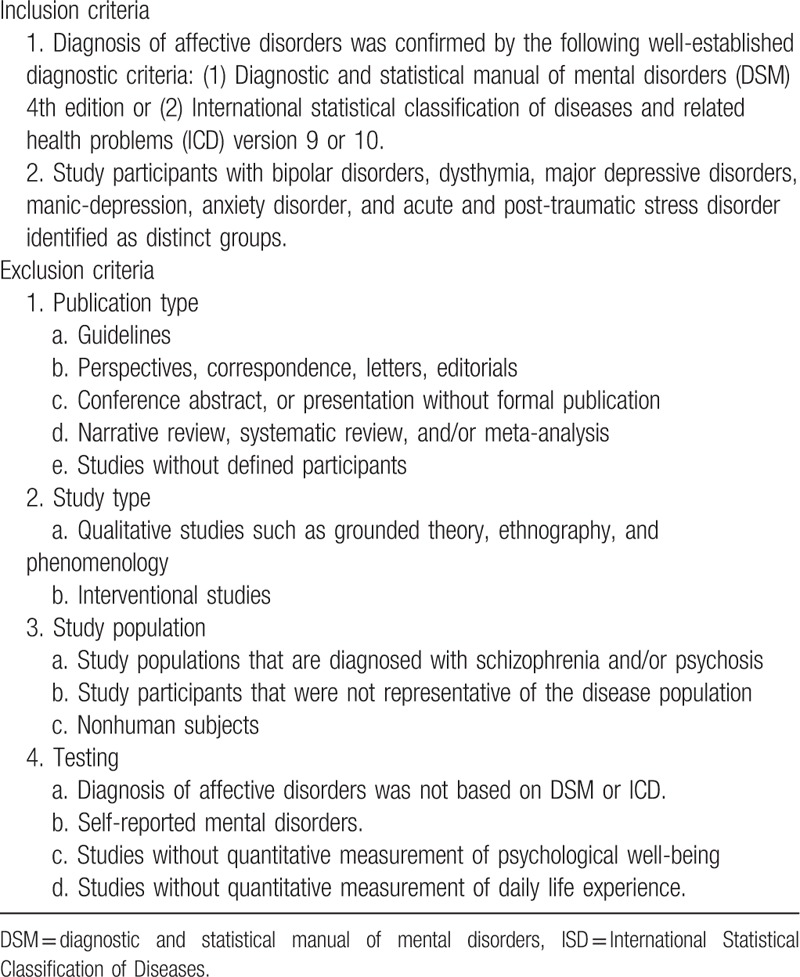
Criteria for selection of studies.

### 2.3 Measurement of exposures and outcomes

Exposures and outcomes are daily life experience and psychological well-being, respectively. Daily life experience can be quantified by validated instruments or their subscales such as hedonic well-being assessment and sickness impact profile (SIP) questionnaire.^[13]^ The SIP comprises 7 modules, which evaluates psychological impact, physical impact, sleep, eating, home management, work, and recreation. Psychological well-being can be measured by World Health Organization Well-being Index and Ryff's scale that quantify the aspects of psychiatric symptoms, self-esteem, and life-satisfaction.^[14]^ Studies using other validated instruments that allow objective quantification of the exposure and outcome will also be included in final qualitative synthesis and meta-analysis.

### 2.4 Quality assessment of the included studies

Quality of the studies will be appraised by the nine-star system of Newcastle–Ottawa scale for nonrandomized observational studies.^[15]^ This assesses subject selection, comparability, and quality of assessment of daily life experience and psychological well-being. Two independent reviewers will rate the studies according to this scale. Inter-rater reliability will be compared using intraclass correlations coefficient (ICC).

### 2.5 Statistical analysis

Studies will be grouped according to distinct mental disorders of the participants.

Summary of effect sizes will be presented as odds ratios (OR), standardized mean difference (SMD) in 95% confidence interval as appropriate. Fisher's *z* transformation will be applied on effect sizes measured as correlation coefficients, weighting by sample size. The weighted average Fisher's *z* will be back transformed to a mean correlation coefficient. Inter-study heterogeneity will be determined using Cochran's *Q* statistics and quantified by *I*^2^ statistics as small, medium, and high degree based on *I*^2^ index of < 25%, >50%, and >75%, respectively.^[16]^ For *I*^2^ more than 50% and *P *< .10 by Cochran's chi-square test, a random effect model will be used to estimate effect sizes in 95% confidence interval. For *I*^2^ less than 50%, a fixed effect model will be employed. The presence of heterogeneity will be further investigated by subgroup analyses and meta-regression as appropriate. Sensitivity analyses will be performed according to the quality of studies. Publication bias will be evaluated by Begg's funnel plot and Egger's test, and corrected by Duval–Tweedie trim-and-fill method. All analyses will be performed using Open Meta-Analyst software, an open access and freely available software developed by the US National Centre for Research Resources and Agency for Healthcare Research and Quality.^[17]^

## 3 Discussion

While stress is unavoidable, sustaining daily life experience may be possible. This will be the first systematic review to demonstrate the association between daily life experience and positive psychological well-being in people living with nonpsychotic mental disorders. Findings of this review will inform prospective interventional trials of enhancing daily life experience in prevention of recurrence and promoting psychological well-being in this population.
